# A Novel Method Applied in Determination and Assessment of Trace Amount of Lead and Cadmium in Rice from Four Provinces, China

**DOI:** 10.1371/journal.pone.0107733

**Published:** 2014-09-24

**Authors:** Shan Li, Mei Wang, Bingyi Yang, Yizhou Zhong, Le Feng

**Affiliations:** Guangdong Key Laboratory of Molecular Epidemiology, School of Public Health, Guangdong Pharmacy University, Guangzhou, China; Tsinghua University, China

## Abstract

Heavy metal contamination of soils or water can lead to excessive lead (Pb) and cadmium (Cd) levels in rice. As cumulative poisons, consumption of Pb and Cd in contaminated rice may cause many toxic effects in humans. In the present study, Pb and Cd levels in rice samples from Hubei, Jiangxi, Heilongjiang, and Guangdong provinces in China were analyzed by cloud point extraction and graphite furnace atomic absorption spectrometry (GFAAS). The heavy metals in the rice samples were reacted with 8-quinolinol to form a complex at pH 9.0 and 40°C. Analytes were quantitatively extracted to a surfactant-rich phase (Triton X-45) after centrifugation and analyzed by GFAAS. The effects of experimental conditions, including pH, concentration of reagents, and equilibration time and temperature, on cloud point extraction were optimized efficiently using Plackett–Burman and Box–Behnken experimental designs. Under the optimum conditions, good linearity was observed in the concentration ranges of 0.5–5 µg/L for Pb and 0.05–0.50 µg/L for Cd. The limits of detection were 0.043 µg/L for Pb with a concentration factor of 24.2 in a 10 mL sample and 0.018 µg/L for Cd with a concentration factor of 18.4 in a 10 mL sample. Twenty rice samples from four provinces were analyzed successfully, and the mean levels of Pb and Cd in the rice were all below their maximum allowable concentrations in China. Comparing the tolerable daily intakes given by FAO/WHO with the mean estimated daily intakes; Pb and Cd mean daily intake through rice consumption were 0.84 µg/kg bw/day and 0.40 µg/kg bw/day, which were lower than the tolerable daily intakes.

## Introduction

Rice is the main staple food in China and a major source of nutrients in the Chinese diet [1]. It contributes 40% of the total calorie intake of Chinese people [2]. With rapid industrial growth in China, there have been reports of high concentrations of heavy metals, such as lead (Pb) and cadmium (Cd), in crops because of soil and water pollution. Cao et al. [3] reported average concentrations of Pb and Cd of 0.054 and 0.014 mg/kg (dry weight), respectively, in rice in the vicinity of an industrial zone in Jiangsu Province, China. Because Pb and Cd enter the human body via ingestion of food [4], the Ministry of Health of the People's Republic of China regulates the maximum levels of Pb and Cd in rice to 0.2 mg/kg [5]. Lead is a cumulative metabolic poison, which acts as a mutagen and teratogen when absorbed in excessive amounts. It has carcinogenic properties, impairs reproduction, liver and thyroid functions, and interferes with resistance to infectious diseases [6]. Cadmium is also toxic and has a long biological half-life (10–30 years) in the human body. The effects of long-term exposure to small concentrations of cadmium are chronic obstructive pulmonary disease, emphysema, osteoporosis, hypertension, chronic renal tubular damage, and lung, kidney and pancreatic cancer [7].

Because heavy metals are present at low concentrations in the matrices of interest, analysis usually requires pre-concentration of the analyte before detection [8]. Many procedures for pre-concentration of metals, such as solid phase extraction [9], dispersive liquid–liquid extraction [10], homogeneous liquid–liquid extraction [11], immersed single-drop microextraction [12] and cloud point extraction (CPE) [13] have been developed. CPE is becoming an important and practical application of the use of surfactants in analytical chemistry [14]. This technique is based on nonionic surfactants in aqueous solutions forming micelles and increasing turbidity when the solution is heated to a temperature above the cloud point temperature [15]. This method is inexpensive, rapid and can be applied in many fields [16]. Numerous studies have been published describing its theoretical background and proposing extraction and pre-concentration schemes for the determination of organic and inorganic analytes [17]. The CPE methodology has been used for the extraction and pre-concentration of metal ions, after the formation of sparingly water-soluble complexes, as an initial step for their determination by flame atomic absorption spectroscopy [18], graphite furnace atomic absorption spectroscopy (GFAAS) [19], spectrophotometry [20] or flow injection analysis inductively coupled plasma mass spectrometry [21]. The same concept has also been employed for the speciation of metals by flame atomic absorption spectroscopy [22], fluorimetry [23], high-performance liquid chromatography [24] and capillary electrophoresis [25].

Several methods of optimization have been used in metal extraction, but most are difficult and time-consuming, and often require several experiments to be performed and neglect possible interactions between variables. Multivariate techniques for optimization have attracted attention in recent years, because they are rapid, inexpensive, and highly-efficient. The Plackett–Burman design (PBD), which is a two-level fractional factorial design developed by Plackett and Burman, has been used extensively to screen important factors for further investigation [26]. However, PBD does not provide the optimum value for each factor. The Box–Behnken design (BBD) is applied after the PBD to obtain the optimum value for each important variable. The number of experiments (*N*) required for a BBD is defined as *N* = 2*k*(*k*−1)+*C*_0_, where *k* is the number of factors and *C*_0_ is the number of central points [27]. The BBD does not contain combinations for which all factors are simultaneously at their highest or lowest levels, therefore, these designs are useful in avoiding experiments that would be performed under extreme conditions [28].

This paper reports the simultaneous preconcentration of Pb and Cd ions after their formation of complexes with 8-quinolinol in basic media and later analysis by GFAAS, using Triton X-45 as a surfactant. The main emphasis of this work was to optimize the CPE method for the determination of Pb and Cd by PBD and BBD. Using the proposed method, rice samples from four representative provinces, namely Hubei, Jiangxi, Heilongjiang, and Guangdong, in China were analyzed and compared. The results of this study will aid rice market regulation and soil pollution surveys.

## Materials and Methods

### Apparatus

A Z-2000 atomic absorption spectrometer (Hitachi, Tokyo, Japan) including an auto sampler with Zeeman-effect background correction was used. A hollow cathode lamp (7.5 mA lamp current and 1.3 nm slit-width) was used as the radiation source for Pb and Cd at wavelengths of 283.3 and 228.8 nm, respectively. The injection volume was 20 µL. The furnace programs for GFAAS are listed in Table 1. A DK-600 thermostated bath (Shanghai Precision Experimental Equipment Co. Ltd., Shanghai, China) was used to maintain the desired temperature, and phase separation was assisted using a centrifuge (Anke KA-1000; Shanghai Anting Scientific Instrument Factory, Shanghai, China). The pH values were controlled with a PHS-3C precision pH meter (Shanghai Hongyi Instrumentation Co., Ltd., Shanghai, China). A MDS-2003 microwave digestion system (Shanghai Xinyi Microwave Chemistry Technology Co., Ltd., Shanghai, China) was used to digest samples.

**Table 1:** Furnace programs.

Step	Start Temp/°C		End Temp/°C		Ramp Time/s	Hold Time/s	Gas Flow l/min
	Pb	Cd	Pb	Cd			
Drying	80	80	140	140	40	0	200
Pyrolysis	400	400	400	600	20	0	200
Atomization	2000	1500	2000	1500	0	5	30
Cleaning	2200	1800	2200	1800	0	4	200

### Reagents

All reagents used in this work were of at least analytical purity grade. Ultrapure water was prepared using a SZ-97 automatic triple water distiller (Shanghai Yarong Biochemical Instrument Factory, Shanghai, China). Working solutions of Pb and Cd were prepared by serial dilution with ultrapure water from stock solutions (Pb 1000 mg/L, and Cd 1000 mg/L, Beijing Nuclear Industry Institute of Chemical Metallurgy, Beijing, China). The following solutions were prepared: 8-quinolinol (1%, m/v) in ethanol, Triton X-45 (5.0%, v/v) in water, and HNO_3_ (1%, v/v) in methanol.

All laboratory glassware used for trace analysis was kept in 5% (v/v) nitric acid for at least 24 h, and then washed several times with ultrapure water before use.

### CPE procedure

Each CPE was carried out according to the following procedure. An aliquot (10 mL) of a solution containing Pb(II) (1 µg/L) or Cd(II) (0.1 µg/L), 0.5 mL of 5% Triton X-45, 0.3 mL of 1% 8-quinolinol and 2.0 mL of buffer solution (pH 9.0) was left in a thermostated bath at 40°C for 15 min. Separation of the phases was achieved by centrifugation at 1645 g for 10 min. The whole system was cooled in an ice-bath for 10 min to increase the viscosity of the surfactant-rich phase. Then the supernatant aqueous phase was carefully removed with a pipette and the surfactant-rich phase was diluted to 0.3 mL with 1% HNO_3_ in methanol to reduce its viscosity. The dilute surfactant-rich phase was introduced into the GFAAS for analysis.

### Optimization procedure

A PBD was used to screen eight factors for those that significantly affected the CPE. The eight factors were pH, concentration of 8-quinolinol, concentration of Triton X-45, equilibration temperature, equilibration time, centrifugation speed, centrifugation time, and cooling time. A BBD was employed in further study to obtain the optimum value for each significant factor. Both experimental designs were carried out with SAS 9.0 (SAS Institute, Cary, NC). The PBD and BBD data were evaluated using the REG and RSREG procedures of SAS 9.0, respectively.

### Analysis of rice samples

Twenty-five rice samples were collected from markets in four Chinese provinces in December, 2013. Rice samples were washed in ultrapure water and oven-dried at approximately 40°C for 24 h. Then the rice samples were ground in a mill and stored at 4°C. About 0.1 g of each rice sample or certified reference material GBW 10045(rice)(Institute of Geophysical and Geochemical Exploration, China) was weighed and digested with 5 mL of HNO_3_ and 1 mL of HClO_4_. Table 2 shows the operating conditions for microwave digestion. After digestion, the digest were transferred to flasks and concentrated to 0.5–1 mL by heating to remove HNO_3_ and HClO_4_ gas completely. The solutions were then transferred to test tubes and diluted to 10 mL with ultrapure water. The solutions were prepared for analysis of Pb and Cd.

**Table 2:** Microwave digestion conditions.

step	Power(W)	Pressure(M Pa)	Time(min)
1	400	0.3	3
2	400	0.6	2
3	600	0.9	2
4	800	1.2	5

## Results and Discussion

### PBD

The PBD was employed for screening the most significant factors affecting the extraction efficiencies of Pb and Cd. In the CPE produce, there are eight factors that may influence the extraction efficiency. These factors are the pH, concentrations of 8-quinolinol and Triton X-45, equilibration temperature and time, centrifugation speed and time, and cooling time. Both the minimum (−) and the maximum (+) levels for these factors were selected for an aqueous solution containing 1.0 µg/L Pb or 0.1 µg/L Cd. Each independent variable was tested at two levels, high and low, denoted by +1 and −1, respectively. The experimental design is detailed in Table 3.

**Table 3:** Levels of the variables and statistical analysis of PBD.

Factors	Code	Low level	High level
pH	x_1_	6.0	10.0
8-quinolinol (m/v, %)	x_2_	0.01	0.04
Triton X-45(v/v, %)	x_3_	0.1	0.3
equilibrium temperature(°C)	x_4_	25	40
equilibration time (min)	x_5_	5	20
centrifugation speed (g)	x_6_	1375	1939
centrifugation time (min)	x_7_	5	15
Cool time	x_8_	5	15

**Figure 1:**
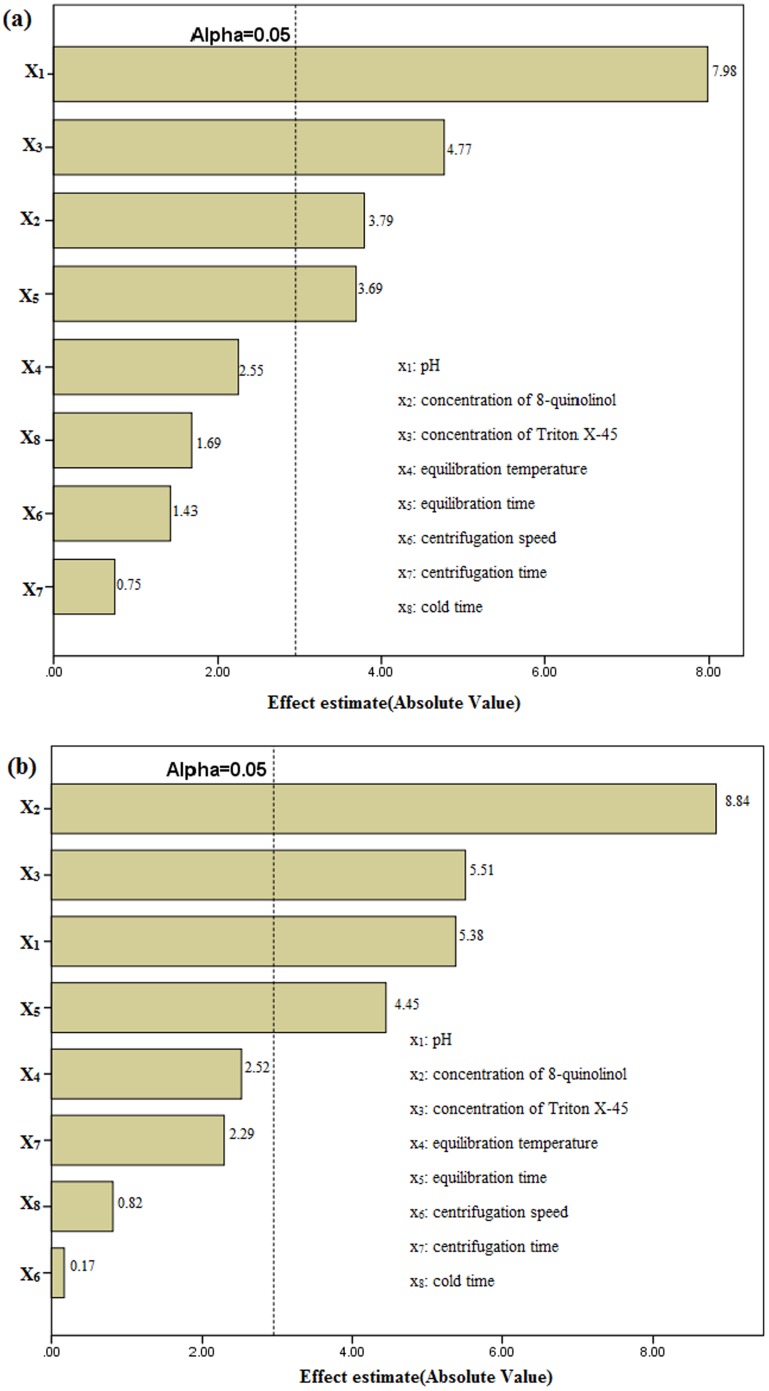
Pareto chart of the PBD results for the analysis of the variables for Pb (a) and Cd (b).

Based on statistical analysis, Figure 1 shows the effects of these components on the response and the significance levels. The effects of 8-quinolinol concentration, Triton X-45 concentration, pH and equilibration time all had significance levels above 95% for Pb and Cd. These factors were identified as having a significant influence on the extraction efficiency. The other factors investigated had no obvious effects and low confidence levels, and were considered insignificant. In the results, the R^2^ were 0.9766 for Pb and 0.9826 for Cd, which means that the model could explain 97.66% (Pb) and 98.26% (Cd) of the total variance in the system. A more detailed evaluation was required to study the effect of interaction between variables, and the variables with confidence levels above 95% were used for further optimization.

### BBD

**Figure 2:**
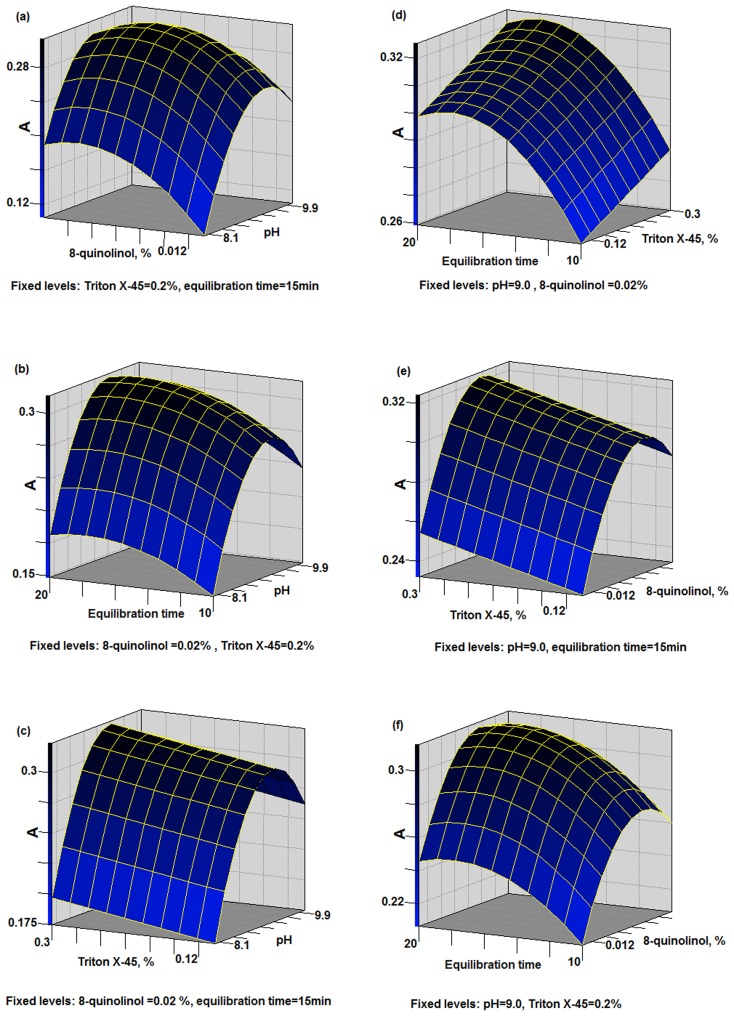
Response surfaces result from BBD for Pb.

According to the results of the PBD, four variables with significant effects on the absorbance were further optimized using a response surface BBD. These factors were the pH, concentrations of 8-quinolinol and Triton X-45, and equilibration time. The design included 27 experiments, and each parameter was tested at three levels in multiple combinations (Table 4). The responses obtained using SAS 9.0 are shown in Fig. 2. The variables that were shown to be insignificant by the PBD were given fixed values as follows: equilibrium temperature (35°C), centrifugation speed (1645 g), centrifugation time (10 min) and cooling time (10 min).

**Table 4:** BBD with three independent variables.

Code	pH	8-quinolinol/%, m/v	Triton X-45/%, v/v	equilibration time/min
	x_1_	x_2_	x_3_	x_4_
−1	8	0.01	0.10^a^/0.15^b^	10
0	9	0.02	0.2	15
1	10	0.03	0.3^a^/0.25^b^	20

a For Pb,

b For Cd.

The R^2^ value for the model of Pb was 0.9672, which indicated that 96.72% of variation observed in the absorbance of Pb could be attributed to the four investigated factors. For Cd, the R^2^ value for the model was 0.9212. The F value of the model for Pb was 27.23 (p<0.05), while the F value for Cd was 12.14 (p<0.05). This indicated that the models for Pb and Cd were significant. For Pb, the values of ‘probability >F′ less than 0.05 indicated x_1_, x_2_, x_3_, x_4_, x_1_*x_1_, x_1_*x_2_, x_2_*x_2_ and x_4_*x_4_ were the model terms which significantly influenced absorbance of Pb. For Cd, x_1_, x_2_, x_3_, x_4_, x_1_*x_1_, x_1_*x_2_ and x_2_*x_2_ were the model terms which significantly influenced absorbance. The lack of fit F values of 7.7035 (Pb) and 12.15 (Cd) showed that lack of fit was insignificant.

**Figure 3:**
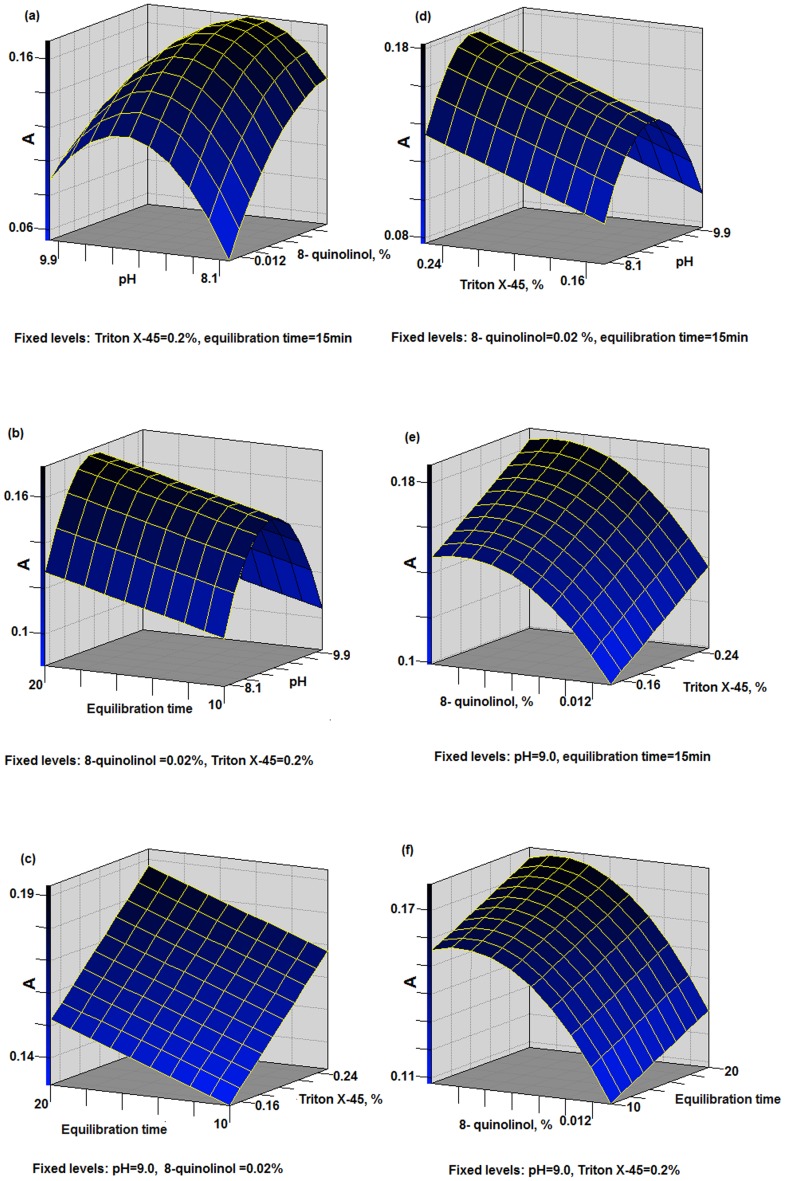
Response surfaces from BBD for Cd.

Figure 2 (a–f) shows the response surfaces obtained for Pb after CPE, while Fig.3 (a–f) shows the response surfaces for Cd. The pH and concentration of 8-quinolinol were important parameters. At pH values of 8.0 and 10.0, the absorbance was significantly lower than at other pH values, probably because the lead ions could not form complexes efficiently with 8-quinolinol at pH 8.0 or 10.0 (Fig. 2 (a–c)). With a low concentration of 8-quinolinol, the extraction efficiency was poor, because there was not sufficient 8-quinolinol to form complexes with all of the Pb ions (Fig. 2 (a, e, f)). These results indicate considerable interaction between the pH and concentration of 8-quinolinol. The response curve was maximized at around pH 9.2 and an 8-quinolinol concentration of around 0.22% (Fig. 2 (a)). After the pH and 8-quinolinol concentration, the equilibration time was also important for determining the CPE efficiency (Fig. 2 (d, b, f)). The maximum extraction efficiency was obtained when the equilibration time was between 16 and 18 min for Pb(II). Decreasing or increasing the equilibration time outside of this range resulted in the extraction efficiency of Pb(II) decreasing. The only parameter that did not have a significant influence on the response was the concentration of Triton X-45. Excess surfactant should decrease the signal because it will increase the overall analyte volume, but this may not have happened in this case because the Triton X-45 was not concentrated enough.

The response surfaces obtained for Cd (Fig. 3) showed similar behavior to Pb. The pH and concentration of 8-quinolinol were significant (Fig. 3(a)), which agreed with the PBD results. The maximum absorbance was obtained at around pH 8.7 and 0.26% (m/v in ethanol) 8-quinolinol, indicating an interaction between these factors. Although the equilibrium time is a key parameter for Cd, unlike that observed for Pb, a quadratic relationship between equilibrium time and absorbance is not found (Fig.3(b, c, f)), and no changes were observed in the absorbance between 17.5 and 20 min (Fig.3 (b, c, f)). Fig.3(c–e) shows that the concentration of Triton X-45 also did not reveal a quadratic term influence, as was observed for Pb.

The experimental design results showed the optimized conditions for Pb were pH 9.1, 0.022% 8-quinolinol, 0.26% Triton X-45, and 17 min equilibration time. For Cd, the optimized conditions were pH 8.7, 0.026% 8-quinolinol, 0.15% Triton X-45 and 18 min equilibration time. The optimization of process parameters using the two-step approach with PBD and BBD was successful.

### Effect of coexisting ions

The effects of common coexisting ions on the CPE of Pb and Cd were investigated. In these experiments, an aliquot of an aqueous solution containing either 1 µg/L Pb or 0.1 µg/L Cd and coexisting ions was analyzed according to the proposed procedure. The tolerance limits for coexisting ions were determined as the coexisting ion concentrations that gave an error of less than a 5% for the determination of Pb and Cd. For 1 µg/L Pb, the results were 1∶5000 for K^+^ and Na^+^, 1∶2000 for Fe^3+^, Mg^2+^ and Al^3+^, 1∶1500 for Ca^2+^, 1∶500 for Zn^2+^, and 1∶25 for Cd^2+^. For 0.1 µg/L Cd, the results were 1∶5000 for K^+^ and Na^+^, 1∶2000 for Mg^2+^, 1∶1000 for Ca^2+^ and Al^3+^, 1∶500 for Fe^3+^ and Zn^2+^, 1∶20 for Pb^2+^.

### Method validation

The limit of detection of the proposed method was studied under the optimal experimental conditions using blank solutions. The detection limits of the investigated elements based on three times the standard deviation of the blank (*n* = 6) were 0.043 µg/L for Pb and 0.018 µg/L for Cd.

The reproducibility of the presented procedure was evaluated (*n* = 7). The relative standard deviations were 0.6% (Pb) and 0.57% (Cd). The linear range for Pb was 0.5–5 µg/L and the regression equation was A = 0.2235*c* ( µg/L) +0.0323 with a correlation coefficient of 0.9992 and an enhancement factor of 24.2. The regression equation for Cd was A = 0.9021*c* (µg/L) +0.0201 (the linear range was 0.05–0.50 µg/L, r = 0.9991) and the enhancement factor was 17.2.

### Analysis of Pb and Cd in rice samples

The proposed method was employed for the determination of Pb and Cd in rice samples. For each rice sample, 6 mL of the sample solution was preconcentrated by the proposed method. Spike recovery experiments for Pb and Cd were carried out for three samples. The results are shown in Tables 5 and 6. The recoveries for Pb and Cd spiked in the rice samples studied were calculated to be in the range of 95.7–104.1%, indicating no interference encountered from these sample matrices. In order to further validate the method for accuracy and precision, a certified reference material GBW 10045 (rice) was analyzed. The obtained values for Pb were 0.068±0.021 mg/kg (*n* = 5) and for Cd were 0.187±0.03 mg/kg (*n* = 5) respectively, which were in good agreement with certified values 0.070±0.023 mg/kg (Pb) and 0.190±0.02 mg/kg (Cd). The mean concentration of Pb was 0.1555 mg/kg (range 0.0622–0.1952 mg/kg), while that of Cd was 0.0736 mg/kg (range 0.0200–0.1232 mg/kg). Compared with the maximum allowable concentration (MAC) recommended by Chinese legislation, all samples contained lower levels of Pb and Cd. These results are similar to those of Chen et al. [29] and Zhu et al. [1]. In the present study, the level of Cd in rice from Heilongjiang province was significantly (p<0.01) lower than that in rice from other provinces, but the Pb level was not significantly different. The main factor leading to the different levels of Cd is genotype difference. Generally, Cd accumulation in shoots and grains are potentially higher in *indica* cultivars compared to *japonica* cultivars, moreover, some specific cultivars among *indica* rice accumulate high level of Cd in vegetative tissues and grains [30]. In rice, there are seven Nramp genes. Takahashi [31] has established that OsNRAMP1 is one of crucial proteins responsible for higher Cd accumulation in rice. The level of OsNRAMP1 expression in the roots of *indica* cultivars was higher than in *japonica* cultivars [32]. Therefore, differences in the expression levels of OsNRAMP1 may be responsible for the observed differences in Cd accumulation among these cultivars.

**Table 5:** Analysis of rice samples and recoveries (*n* = 3).

Sample	Cd			Pd		
	Found (µg/g)	Added (µg/g)	Recovery (%)	Found (µg/g)	Added (µg/g)	Recovery (%)
Heilongjiang 2	0.0200	0	-	0.1693	0	-
	0.0394	0.020	97.0	0.3320	0.170	95.7
Jiangxi 2	0.0771	0	-	0.0622	0	-
	0.1598	0.080	103.4	0.1208	0.060	97.7
Jiangxi 3	0.0490	0	-	0.1041	0	-
	0.0986	0.050	97.2	0.2082	0.100	104.1

**Table 6:** Analysis of rice samples from four Chinese provinces (mg/kg).

Provinces	Cd a		Pb	
	Range	Mean±SD	Range	Mean±SD
Hubei	0.0833–0.1232	0.1047±0.0194	0.1400–0.1673	0.1539±0.0107
Heilongjiang	0.0200–0.0359	0.0290±0.0068	0.1452–0.1866	0.1715±0.0160
Jiangxi	0.0490–0.1089	0.0861±0.0240	0.0622–0.1737	0.1329±0.0483
Guangdong	0.0661–0.0944	0.0747±0.0112	0.1265–0.1952	0.1637±0.0265

a P<0.01.

Based on the dietary nutrition intake level survey by Zhong et al. [33], rice was the staple food for daily consumption, and the adult had an average daily intake of 323 g rice per day. The adult body weight was set to 60 kg in this study. The mean intakes of Pb and Cd through rice are estimated to be 0.84 µg/kg bw/day (range 0.34–1.05 µg/kg bw/day) and 0.40 µg/kg bw/day (range 0.11–0.66 µg/kg bw/day) for adults. Comparing with the tolerable daily intake (TDI) [34] set by the Joint FAO/WHO Expert Committee on Food Additives (JECFA) for Pb at 3.6 µg/kg bw/day and Cd at 1 µg/kg bw/day, the maximum daily intakes of Pb and Cd for adults were within the safe limit. It indicates that the longterm large consumption of rice may not result in the high exposure of Pb and Cd. Although the results in this work were positive, intake of Pb and Cd from other food stuff should also be of concern.

## Conclusion

A novel non-chromatographic speciation technique using sequential cloud point extraction was developed for determination of Pb and Cd by GFAAS. This method permits rapid and accurate direct determination of Pb and Cd in rice. This method is inexpensive and has good reproducibility. Applying this technique, rice samples from four Chinese provinces (Hubei, Heilongjiang, Jiangxi, and Guangdong) were analyzed. The mean levels of Pb and Cd in rice were all below the Chinese MAC for these metals. The maximum daily intakes of Pb and Cd for adults were lower than tolerable daily intake (TDI) given by FAO/WHO.
